# Improving Dynamic Material Characterization in SHPB Tests Through Optimized Friction Correction

**DOI:** 10.3390/ma18184327

**Published:** 2025-09-16

**Authors:** Alexis Rusinek, Tomasz Jankowiak, Amine Bendarma

**Affiliations:** 1 Laboratory of Microstructure Studies and Mechanics of Materials (LEM3), Arts et Métiers Institute of Technology, Lorraine University, UMR CNRS 7239, 57073 Metz, France; alexis.rusinek@univ-lorraine.fr; 2 Institute of Structural Analysis, Poznan University of Technology, Piotrowo 5, 60-965 Poznań, Poland; 3 Arts et Métiers Campus de Rabat, Technopolis, Rocade de Rabat-Salé, Sala Al Jadida 11100, Morocco; amine.bendarma@artsetmetiers.ma; 4 L’Université Privée de la Santé et des Sciences d’Agadir (UPSSA), CC9X+3M3, Agadir 80000, Morocco

**Keywords:** SPHB, dynamic compression test, numerical simulation, geometries, friction correction

## Abstract

This study examines the influence of friction at the specimen–bar interface on the macroscopic response of materials during dynamic compression tests using the split Hopkinson Pressure Bar (SHPB) under high-deformation-rate conditions. A mesoscale model is employed to simulate and compare results with experimental data, and a finite element model of cylindrical specimens with varying slenderness ratios is developed in Abaqus/Explicit. Numerical analyzes show that both specimen geometry and boundary conditions, particularly friction, have a decisive impact on the accuracy and reliability of SHPB measurements. A friction correction method based on barreling factor and plastic deformation demonstrates closer agreement with experimental observations than conventional approaches, revealing that the widely used Avitzur model may overestimate friction by 34–39%. The results highlight the importance of accurate friction correction and the selection of optimal specimen dimensions to minimize testing errors. These findings improve the precision of dynamic material characterization and support the development of more reliable constitutive models to predict material behavior across a broad range of strain rates.

## 1. Introduction

**Figure 1:**
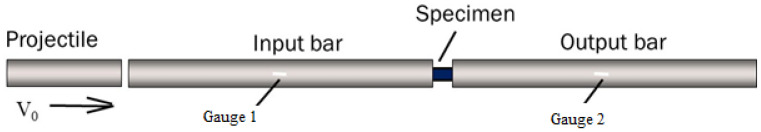
Schematic representation of the SHPB for dynamic compression.

Dynamic compression testing employing Hopkinson pressure bars, commonly referred to as Split Hopkinson Pressure Bars (SHPBs), is a well-established and widely used methodology to characterize the behavior of materials under high strain rate conditions [1,2,3,4]. This method, first developed by Hopkinson and later refined, provides valuable insight into the mechanical response of materials subjected to rapid loading conditions. The SHPB setup consists of an input bar, an output bar, and a projectile (a striker bar), Figure 1. A stress wave generated by the impact of the striker bar propagates through the input bar, interacts with the specimen placed between the input and output bars, and transmits valuable information about the material’s dynamic properties.

One of the critical aspects of SHPB testing is the influence of the geometry of the specimen on the observed material behavior. Among the various geometric factors, the height-to-diameter ratio (*H*/*D* ratio) of the samples plays an important role. This ratio can significantly affect the accuracy and reliability of the measured dynamic stress–strain response. For accurate results, it is essential to maintain a balance between the dimensions of the specimen to minimize errors arising from wave dispersion, inertial effects, and friction. In addition, a low ratio *H*/*D* (short and wide specimens) tends to reduce axial strain localization and helps to achieve a more uniform stress distribution across the sample cross section. However, this configuration can lead to significant radial inertia effects and potential barreling of the specimen, which complicates the interpretation of the results. In contrast, a high ratio *H*/*D* (tall and slender specimens) minimizes radial inertia and barreling, but increases the likelihood of axial strain gradients and premature failure modes, such as buckling. The specimen height *H* also affects the average strain rate and the final strain achieved during dynamic tests. These scale effects must be carefully considered to ensure the validity of experimental data. Barreling is caused by friction. At the same time, the stress state in the specimen during compression is no longer uniaxial, which increases the stress level compared to the uniaxial state. Eliminating the effect of friction is therefore essential [2,3]. Friction correction has been highlighted as crucial in many studies. Researchers have also attempted to reduce friction effects by using specific specimen shapes, such as rings [5] or bone-shaped samples [3]. Finally, the finite element method is combined with experiments to obtain friction calibration curves.

The friction effect is important during the testing of various materials such as concrete [6,7] or calcium silicate [8]. The results showed that frictional constraints had a strong influence on the maximum stress value and the softening phase. Uniaxial compression tests are crucial for assessing concrete strength, essential in structural design [9]. Friction at the platen–specimen interface can have an impact on results, altering failure mechanisms, and inflating strength. The study explores friction effects on cylindrical specimens with varying slenderness, using a mesoscale model to simulate and compare scenarios with experimental data.

Friction at the interface between the compression die and the specimen has a critical influence on metal-forming processes [10]. The authors examine the friction coefficient during compression loading of the aluminum alloy AA6060 and steel 42CrMo4 under varying lubrication conditions. The tests used 4 mm cylindrical specimens with *d*_0_/*h*_0_ ratios of 0.5, 0.67, 1.43, and 2.0. Lubrication setups included dry, Molykote, single Teflon layer, and two Teflon layers with an oil film. Friction coefficients were calculated from deformation resistance curves relative to *d*_0_/*h*_0_ values at different strains and lubrication conditions. Flow curves were compared after accounting for friction effects across all *d*_0_/*h*_0_ ratios. The barreling compression test is a vital method for characterizing the mechanical behavior of deformed materials and analyzing interfacial friction using Avitzur’s “limit analysis of disc and strip forging.” While effective under low friction conditions, this method requires deeper evaluation [11]. The study extensively examines the Avitzur model, exploring its applicability and limitations. The model’s kinematics are detailed to describe the barreling parameter and deformed sample profile. The analysis identifies a valid method for evaluating friction factors up to 0.5.

Dynamic compression testing using SHPB has undergone significant development since its inception, focusing on the resolution of issues related to specimen geometry, boundary conditions, and wave propagation effects. Recent SHPB techniques have placed a strong emphasis on the requirement of dynamic stress equilibrium during tests [12]. The work of Song and Chen first highlighted that the establishment of a stress equilibrium early in the test is a prerequisite for valid mechanical property characterization, particularly for low-wave impedance materials.

Frew et al. [13] presented analytical models and experimental techniques for obtaining dynamic stress–strain data in compression of elastoplastic materials. It describes a modification of the SHPB (Split Hopkinson Pressure Bar) apparatus using copper and steel pulse shapers to obtain a dynamic stress equilibrium and a constant strain rate. Models predicting incident stress pulses are developed and experimentally validated. The study focuses on 4340 Rc 43 steel, demonstrating the importance of pulse shaping for reliable results. The experimental results show good agreement with the model predictions.

The effect of the geometry of the specimen, particularly the height-to-diameter (*H*/*D*) ratio, has been well reported in the literature. Ramo et al. [14] showed that inappropriate specimen dimensions could lead to reflections of stress waves and errors in the stress-strain curve, particularly for brittle materials. Furthermore, boundary conditions, such as friction at the interfaces between the specimen and the bars, are vital to the accuracy of experimental results. Zhou et al. [15] investigated the effect of friction on the homogeneity of strain and indicated that lubrication and proper surface preparation could alleviate these effects. Pulse shaping has emerged as an influential technique to refine the quality of SHPB tests. Vecchio et al. [16] demonstrated that pulse shaping enhances stress uniformity and decreases high-frequency oscillations; thus, it allows accurate control over the strain rate subjected to the sample. This technique is particularly useful when dealing with materials sensitive to strain rate variations; hence, more reliable and repeatable results are achieved.

Within this important domain of material properties under high strain rates, the transition from isothermal to adiabatic conditions during deformation is a significant factor. Jankowiak et al. [2] investigated this phenomenon with attention to the coupling between thermal and mechanical effects on material behavior. Their work flags the requirement for advanced methods of testing in which temperatures are taken into account as changes occur in the course of a rapid loading. In addition to the experimental work, further developments in numerical modeling have, so to speak, given more insight into the SHPB test mechanics. In terms of optimizing configurations and exploring complex stress states within a sample, finite element simulations, such as those by Ubertalli et al. [17], surely have contributed a large amount. Such models help to bridge the gap between experimental observations and theoretical predictions, thus increasing our comprehension of material behavior under dynamic loading.

Accurate determination of material properties under compressive forces is often obscured by unwanted side effects, such as friction, specimen inertia, and localized triaxial stress states. Recent work by Jankowiak et al. [3] meets these challenges with the introduction of a new sample geometry that aims to reduce the influence of such effects to a minimum. Based on this, a comprehensive framework for analyzing and mitigating such unwanted effects is developed by using finite element simulations under Abaqus/Standard and Abaqus/Explicit. This approach enables the correlation of macroscopic measurements, such as stress and strain, with localized values in the material that are not usually accessible during empirical studies. The study also introduces correction equations for the friction and triaxial stress states so that a more detailed understanding of the experimental data can be achieved. The new approach also enables determination of the failure strain as it relates to stress triaxiality, a parameter very important for applications that demand reliable material modeling at any strain rate. These advances encourage the reliable and precise characterization of material responses, especially under dynamic loading conditions. This kind of specimen is also used in the new SHPB testing procedure of the material at high temperatures to reduce the heating of the bars [18].

The dynamic mechanical response of materials is highly sensitive to these geometric parameters, which requires a systematic study to optimize the *H*/*D* ratio for various types of materials and loading conditions. Recent advances in SHPB techniques and high-speed diagnostics have enabled more precise control and measurement of these dynamic tests, allowing a deeper understanding of the underlying behavior of the material [2].

Boland et al. [19] have designed a new type of Split Hopkinson Pressure Bar (SHPB) for testing dynamic behavior in adhesively bonded joints at high strain rates. Research is deemed essential primarily for industries related to automotive and aerospace due to the considerable static and dynamic loads experienced by the bonded joints. The machine is equipped with a pneumatic actuator capable of operating at speeds up to 30 m/s and a lever-operated braking system to stop the actuator under tensile and compressive testing. This work provides a detailed description of the functionalities of the machine, including tensile and compressive pressure bars configurations, thus being a valuable tool for assessing the performance of adhesive joints under impact conditions.

This achievement emphasizes the importance of impact testing for adhesive joints to allow accurate numerical modeling and understanding of the mechanical behavior given by the bonded joining in the structural field.

Tarfaoui et al. [20] will investigate the optimization of the Split Hopkinson Pressure Bar (SHPB) system for the dynamic characterization of composite materials at high strain rates. Numerical investigations of alternative bar geometries are presented—square, hexagonal, and triangular cross-sections—in comparison with conventional cylindrical bars. A validated 3D finite element model for cylindrical bars is used as a reference to analyze the performance of the proposed geometries. These alternative geometries bring some remarkable advantages, such as in situ imaging of specimen side surfaces during stress wave propagation and improved bonding of strain gauges under high impact pressure. All the results show excellent consistencies between the dynamic behavior of the specimens and the bar geometries, which validate that tailoring SHPB systems to the specific testing requirements of composite materials is appropriate.

Specifically, the specimen height-to-diameter *H*/*D* ratio has been found to have a significant effect on wave propagation, stress equilibrium, and inertial effects [21]. In the same manner, boundary restraints at the contact interface result in frictional forces that change the state of stress, sometimes causing premature localization or incorrect interpretation of the stress-strain behavior [22]. Even though numerous investigations have tried to compensate or simulate these effects, there is still a lack of thorough numerical investigation examining the combined effect of geometry and boundary friction. The present study fills this shortage by adopting a finite element method to examine the effects of specimen geometry and interfacial boundary conditions on SHPB result reliability.

In general, continued refinement of SHPB methodologies, combined with advances in numerical modeling and pulse shaping techniques, has significantly expanded their applicability. These developments enable researchers to characterize a wide range of materials, from metals to brittle composites, under extreme loading conditions.

Friction in compression tests has been widely studied in the literature. Among the classical approaches, Avitzur (1969) stands out, as it provides an analytical expression relating the barreling of the specimen to the friction coefficient [10,11]. Commonly used in metal forming analyses, this approach allows a more accurate calculation of interface conditions during compression. In the present study, the Avitzur method is applied to determine the friction coefficient based on the final shape of the specimen, ensuring that the observed barreling matches the mechanical behavior of the material.

This study systematically investigates the effects of the *H*/*D* ratio on the dynamic compressive response using SHPB. By examining a range of *H*/*D* ratios, the scale-dependent phenomena are clarified, providing guidelines for selecting optimal specimen dimensions in relation to friction. Friction correction is also addressed. A new model is introduced that links the friction coefficient to specimen deformation, including barreling. These numerical results improve the accuracy of dynamic material characterization and support the development of more reliable constitutive models to predict material behavior under high strain rate conditions.

## 2. Presentation of the Experimental Technique and Quantities Used for Material Description

To define the material behavior at high strain rates and to estimate the strain rate sensitivity of the material, the Split Hopkinson Pressure Bar (SHPB) technique was used, as discussed previously [2]. This study uses a simulation-based approach, employing finite element analysis to examine in detail the results from dynamic compression tests. Two long bars with a diameter of approximately 20 mm are used, with a specimen placed between them, see Figure 1. When the projectile strikes the input bar with an initial impact velocity, an incident wave, $\varepsilon_{I}=\sigma_{I}/E_{b}$, is generated and travels along the bar with a velocity $C_{0}=\sqrt{E_{b}/\rho_{b}}$, where $E_{b}$ is the Young’s modulus of the bars and $\rho_{b}$ is the bar material density. The incident stress intensity (related to the incident strain wave) can be calculated based on Equation (1):

(1) $$\sigma_{I}=\frac{1}{2}\rho_{b}C_{0}V_{P},\;$$

where $V_{P}$ is velocity of the projectile.

To use the theory of elastic waves, it is necessary to stay in the elastic domain via this equation, Equation (2).

(2) $$\sigma_{I}<\sigma_{y}^{SHPB},\;$$

where $\sigma_{y}^{SHPB}$ is the yield stress of the bars.

**Figure 2:**
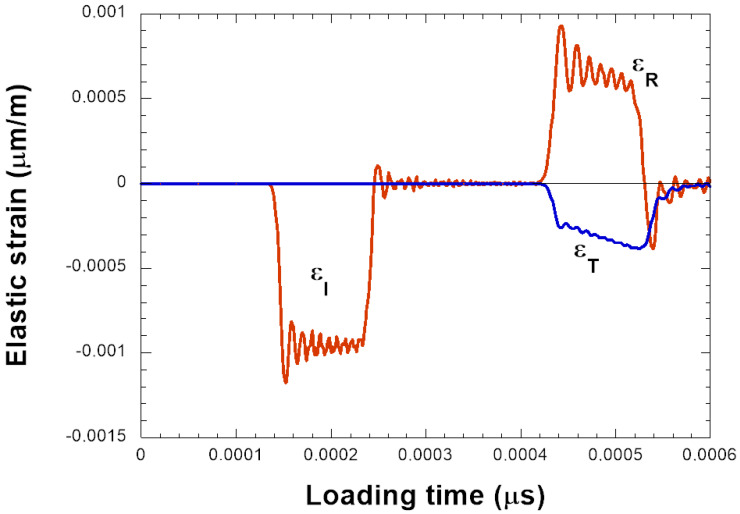
Time evolution of elastic waves during a compression test (numerical simulation).

In our experimental configuration, the yield stress of the bars is equal to $\sigma_{y}^{SHPB}$ = 2 GPa, corresponding to a theoretical maximum impact velocity of $V_{P}^{Max}$ = 100 m/s. However, due to the system used to measure the waves—generally resistance gauges glued on the bars—the maximum impact velocity is limited to $V_{P}^{Exp.Max}$ = 20 m/s. The incident strain associated *ε_I_* is corresponding to 1950 μm/m or to a stress level $\sigma_{I}$ of 400 MPa. A typical signal is reported in Figure 2.

In the previous picture, Figure 2, it is observed that the reflected wave, proportional to the average strain rate $\dot{\varepsilon }$ imposed on the specimen, is not constant in time. The reflected wave decreases as the hardening of the material increases. Therefore, the average strain rate imposed on the material may not be assumed to be constant but decreases with the plastic deformation induced to the specimen. The strain rate is defined as follows Equation (3).

(3) $$\dot{\varepsilon }\left(t\right)=\frac{C_{0}}{H}\left[\varepsilon_{I}\left(t\right)-\varepsilon_{R}\left(t\right)-\varepsilon_{T}\left(t\right)\right],$$

where $C_{0}$ is the celerity of the elastic waves traveling along the bars and *H* is the height of the specimen used.

The other quantities defined during dynamic compression are the force and the displacement imposed to the specimen allowing to calculate the average stress and strain, Equations (4) and (5).

(4) $$\sigma \left(t\right)=\frac{E_{b}A_{b}}{2A_{s}}\left[\varepsilon_{I}\left(t\right)+\varepsilon_{R}\left(t\right)+\varepsilon_{T}\left(t\right)\right],$$

(5) $$\varepsilon \left(t\right)=\frac{C_{0}}{H}\int_{0}^{t_{load}}\left[\varepsilon_{I}\left(t\right)-\varepsilon_{R}\left(t\right)-\varepsilon_{T}\left(t\right)\right]dt,\;$$

where $A_{S}$ and *H* are respectively the cross section area and the height of the specimen. $A_{b}$ is the bars cross section area. These solutions are based on the simplified theory where the elastic waves are traveling along the axial direction.

**Figure 3:**
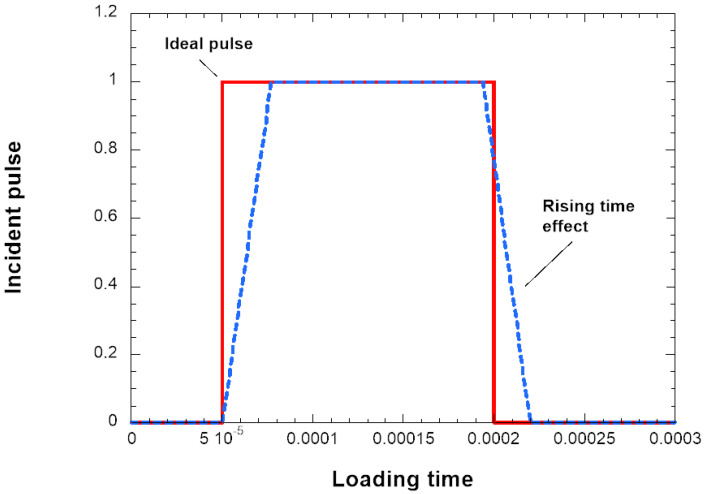
Theoretical signal and case influenced by rising time as during experiments.

However, the signal is not longitudinal, but due to the Poisson effect, induces a radial oscillation of the bars. Using the blue signal described previously with the rising time, since the signal does not go instantaneously from zero to one during impact, the following signal is obtained; see Figure 3.

In addition to the previously described phenomena that affect the measurements, other factors can also change the signal, such as the puncture effect, local plasticity, the flatness of the projectile–bar contact, the position of the gauges, the specimen geometry, and friction. This paper studies how the sample dimensions *H* and *D* and the initial shape factor $s_{0}$ influence the material behavior due to friction.

## 3. Numerical Results and Analysis

**Figure 4:**
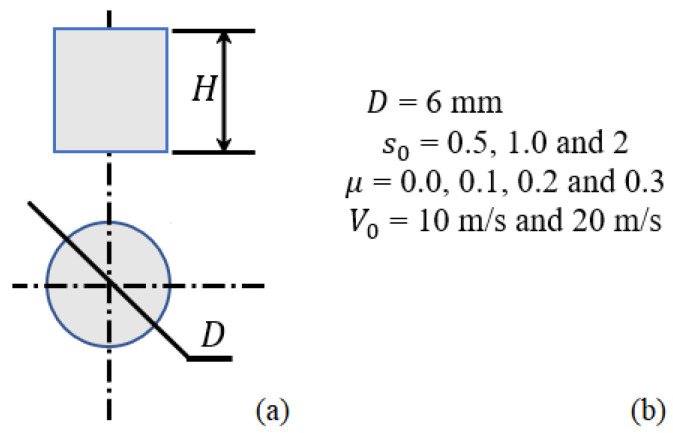
(**a**) Cylindrical specimen in numerical simulations; (**b**) Variables considered in simulations: diameter *D*, shape parameter $s_{0}$, friction coefficient μ, and projectile velocity $V_{0}$.

Numerical models have been built to analyze the different effects, such as shape parameter $s_{0}$ with diameter *D* = 6 mm, friction coefficient μ, and projectile velocity $V_{0}$, on the macroscopic behavior of the material during dynamic compression with Split Hopkinson Pressure Bars (SHPBs). These variables are presented in Figure 4.

**Figure 5:**
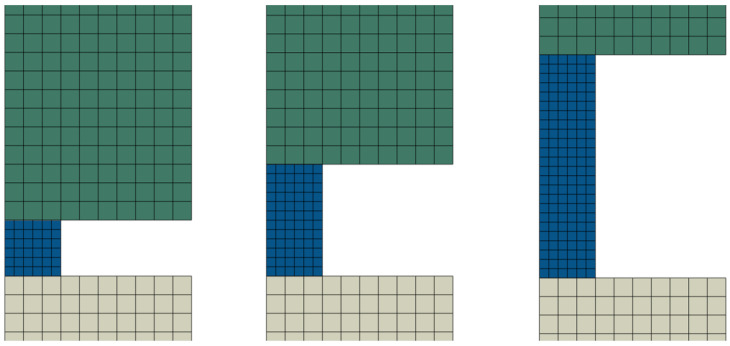
Mesh of the specimen and bars (zoomed) for the geometries used.

The model in Abaqus/Explicit is axisymmetric and contains four parts [23]. All parts are meshed with CAX4R finite elements. The coarse mesh (size = 1 mm) is used in the parts with elastic deformation (projectile, input, and output bars) and the fine mesh (size = 0.5 mm) in part with plastic deformation (specimen); see Figure 5. Appendix A presents the mesh size sensitivity analysis of the results. The contact is considered to occur in between all parts, with an assumed friction coefficient between 0.0 and 0.3. The velocity of the projectile has two values—10 m/s and 20 m/s—and is assigned as the initial velocity of the nodes of the projectile. The total time of the whole dynamic compression process is about 600 μs. Longitudinal stress and strain (22-direction) are recorded during the simulation in the middle of the input and output bar (on the surface in the integration point). In the middle of the specimen on the surface, the same stress and strain are also recorded together with PEEQ (equivalent plastic strain) and von Mises equivalent stress. Thanks to this, the global results obtained based on the three elastic waves and local results recorded directly in the specimen can be compared.

To define the local material behavior, the Johnson–Cook model is used [24,25], including strain hardening and strain rate sensitivity, as seen in Equation (6).

(6) $$\bar{\sigma }=\left(A+B{\bar{\varepsilon }}_{pl}^{n}\right)\left(1+C\ln\frac{{\dot{\varepsilon }}_{pl}}{{\dot{\varepsilon }}_{0}}\right)$$

*A*, *B*, and *n* are the constants related to the yield stress and the strain hardening of the material, *C* is the sensitivity of the strain rate of the material, and ${\dot{\varepsilon }}_{0}$ is the reference strain rate [24]. During numerical simulations, the temperature sensitivity has been omitted.

**Figure 6:**
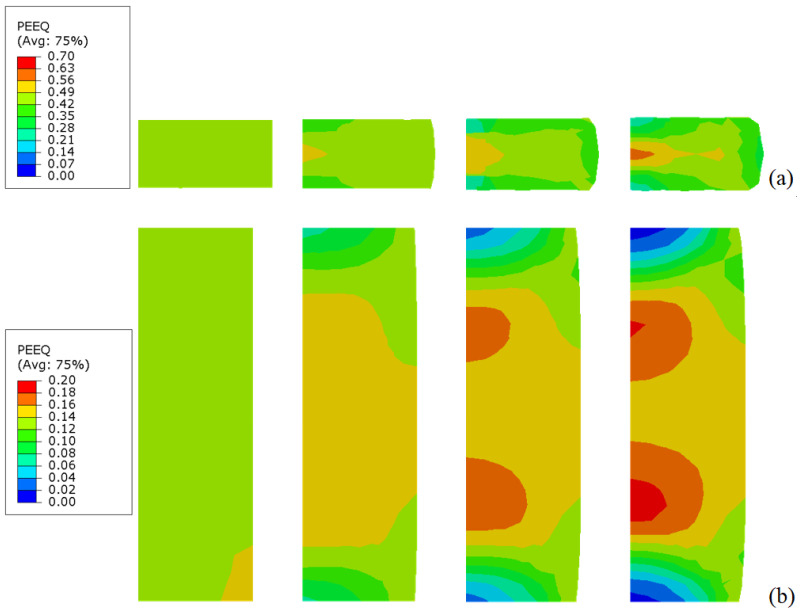
Distribution of the equivalent plastic strain (PEEQ in Abaqus) in the specimen for several friction coefficients (0.0, 0.1, 0.2, and 0.3, from left to right); (**a**) $s_{0}=0.5$ and (**b**) $s_{0}=2.0$.

**Figure 7:**
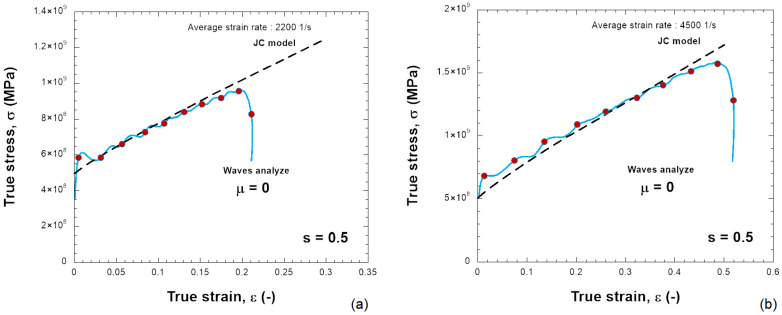
Comparison between the JC model and the behavior obtained with the elastic waves using the following: (**a**) 10 m/s, (**b**) 20 m/s for a friction coefficient of μ = 0.0 and $s_{0}$ = 0.5.

**Figure 8:**
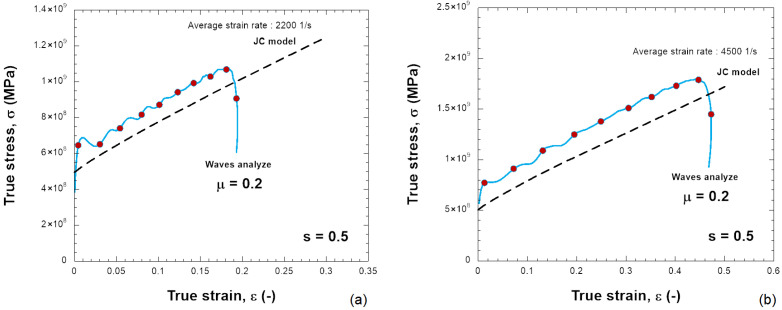
Comparison between the JC model and the behavior obtained with the elastic waves using the following: (**a**) 10 m/s, (**b**) 20 m/s for a friction coefficient of μ = 0.2 and $s_{0}$ = 0.5.

**Figure 9:**
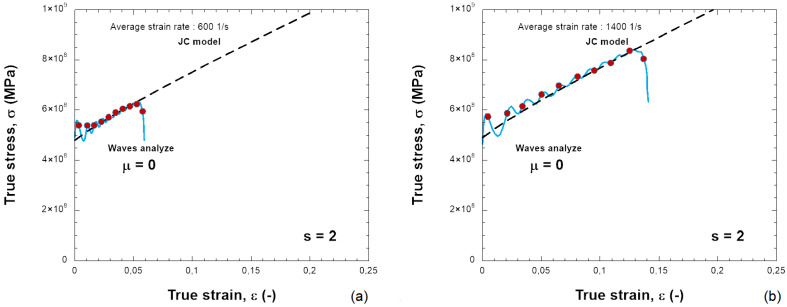
Comparison between the JC model and the behavior obtained with the elastic waves using the following: (**a**) 10 m/s, (**b**) 20 m/s for a friction coefficient of μ = 0.0 and $s_{0}$ = 2.0.

**Figure 10:**
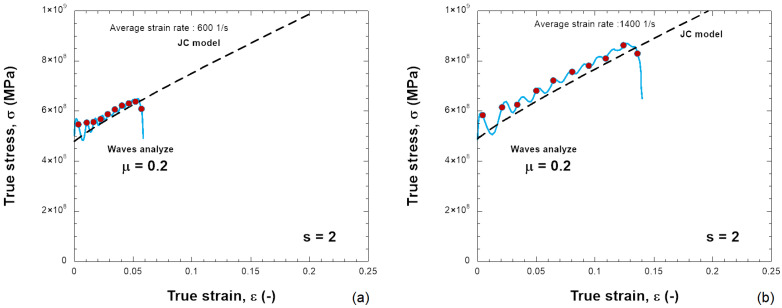
Comparison between the JC model and the behavior obtained with the elastic waves using the following: (**a**) 10 m/s, (**b**) 20 m/s for a friction coefficient of μ = 0.2 and $s_{0}$ = 2.0.

The constants used to describe Inconel 718 behavior are listed in Table 1. It describes a quasi-linear hardening since n→ 1 and a linear strain rate sensitivity. The material used in this work is just an example to show the results of the SHPB test together with the needed friction correction. In the numerical simulations, the specimen is compressed and the plastic strain increases. Figure 6 presents the distribution of PEEQ (equivalent plastic strain) in the samples for various friction coefficients (0.0, 0.1, 0.2, 0.3) and for $s_{0}=0.5$ and $s_{0}=2.0$, with H=6 mm. The barreling increases as the friction coefficient increases. Based on the previous results, the stress–strain curve of the material can be determined, see Figure 7, Figure 8, Figure 9 and Figure 10. The numerical results for different geometries are compared with the Johnson–Cook model, as seen in Equation (6).

For friction μ = 0, the behavior calculated from dynamic compression is in agreement with the input stress–strain curve; see Figure 7a,b and Figure 9a,b for different projectile velocities (strain rates) and for different $s_{0}$. The increase in stress is small and also acceptable with a higher friction coefficient (μ = 0.2) (Figure 10).

**Figure 11:**
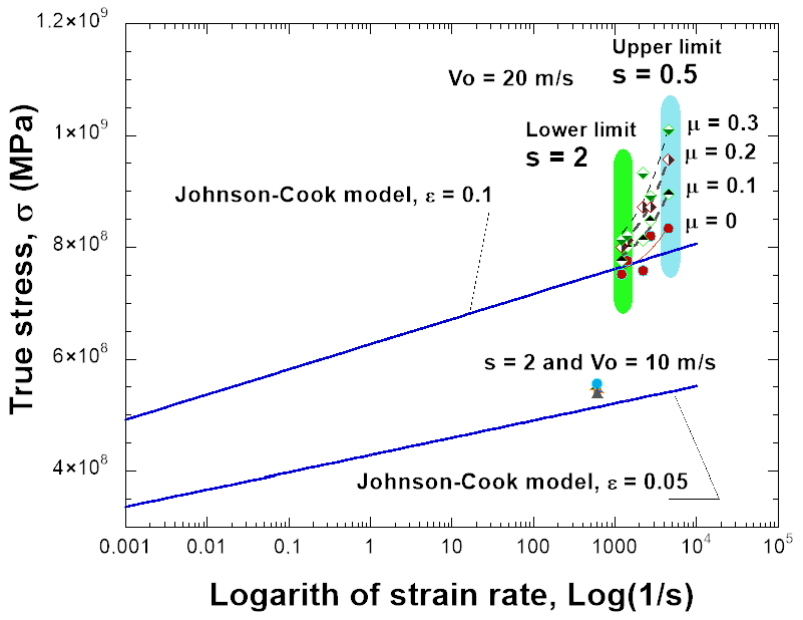
Description of the material behavior using different ratio geometries, friction coefficient, and impact velocity.

If the sample has a smaller shape ratio, $s_{0}=0.5$, the average plastic strain rate increases to ${\dot{\varepsilon }}_{pl}=4400\;s^{-1}$ during the test, and the stress does not match Equation (6), see Figure 8. The overstress increases as the friction at the bar–specimen interface increases. Therefore, it is observed that the relationship of the stress–strain rate of the material is not linear as defined by the Johnson–Cook model but demonstrates artificially a nonlinear sensitivity to the strain rate. The sensitivity to strain rate depends on the shape ratio $s_{0}$ and the friction coefficient μ used (Figure 11). The stress–strain data need to be corrected because the stress is generally higher than the input JC model predicts. The same situation occurs in real experimental tests. By taking barreling and interfacial friction into account, the friction correction improves the accuracy of the average stress-strain curve in the sample.

Figure 11 illustrates the effect of specimen geometry, friction coefficient, and impact velocity on the true stress–strain response obtained from the SHPB simulations. The results show that for slender specimens ($s_{0}=2.0$), the predicted stresses are generally lower and the influence of friction remains moderate across all tested μ values (μ = 0.0, 0.1, 0.2, 0.3). In contrast, for short specimens ($s_{0}=0.5$), the stress response increases significantly, particularly at higher friction levels, highlighting the amplification of barreling and constraint effects. The figure also compares the simulation results with the Johnson–Cook model input behavior for different plastic strain levels (ε = 0.05 and ε = 0.1). The use of a friction correction reduces these discrepancies and allows to determine the real behavior of the material used during experiments.

## 4. Discussion About Friction Correction in Compression Test

Friction plays a critical role in compression testing, as the interaction between the specimen and tooling surfaces (e.g., compression plates or Split Hopkinson Pressure Bars) directly influences the measured response and consequently, the accurate determination of the material’s mechanical properties [2]. Friction causes non-uniform stress distributions, leading to specimen barreling and an overestimation of the deformation force, which can affect the reliability of experimental data. Although lubrication is used to reduce friction, it cannot be completely removed and the actual friction coefficient must always be measured. Equation (7) is used to account for friction effects, but it requires knowing the friction coefficient.

(7) $$\left\{\begin{array}{c} \sigma_{\mathrm{mat}\;}=\sigma_{\mathrm{meas}\;}-\Delta \sigma_{\mathrm{fric}\;}\\ \sigma_{\mathrm{mat}\;}=\sigma_{\mathrm{meas}\;}\left(1-\frac{\mu }{3}\frac{D}{H}\right) \end{array}\right.$$

The newly proposed model complements this approach by enabling a more precise determination of the friction coefficient based on the observed specimen deformation, allowing for improved correction of the stress–strain response, minimization of barreling effects, and enhanced reliability of material characterization. This approach is compared with the model proposed by Avitzur to calculate the friction coefficient.

### 4.1. Friction Correction Using the Avitzur Model

Introduced by Avitzur [10,11], the model applies an upper-bound velocity field to estimate the average deformation pressure in an axisymmetric compression test. It introduces a barreling parameter, typically denoted as *b*, to characterize the curved profile (Bbarreling) of the compressed specimen. The model relates the compression force *F* over the cross-sectional area *S* of the sample to the average stress σ as shown in Equation (8).

(8) $$\frac{F}{S}=\sigma \left(1+\frac{6bH}{R^{2}}\right)$$

Ebrahimi [26] provides a geometric estimate of *b* using the measurable radii and the change in height as follows:

(9) $$b=4\frac{\Delta R}{R}\frac{H}{\Delta H}$$

The symbols are defined as follows: $R_{A}$ is the top radius at the tool–specimen contact, $R_{B}$ is the maximum (bulge) radius, *H* is the final height, and $H_{0}$ is the initial height. The radial increase is given by $\Delta R=R_{B}-R_{A}$, while the height reduction is $\Delta H=H_{0}-H$. Due to compression and friction, the cylindrical shape is distorted such that $R_{A}=R+u_{1}^{\min}$ and $R_{B}=R+u_{1}^{\mathrm{mid}}$. The friction coefficient μ can finally be calculated using the following:

(10) $$\mu_{avit}=\left(\frac{R}{H}\right)b\cdot \frac{3\sqrt{3}}{12-2b}$$

For two cases, $s_{0}=0.5$ and $s_{0}=1.0$, the Avitzur friction coefficient, $\mu_{avit}$, was used to calculate the friction. Two different radii, R=6 mm and R=8 mm, were also considered. The resulting values are 0.095 and 0.159 for H=6 mm (0.104 and 0.169 for H=8 mm), see Table 2. The error for these four cases ranged from 21% to 35%.

The value of friction coefficient μ = 0.078 was assumed for $s_{0}$ = 0.5 and little higher μ = 0.125 for $s_{0}$ = 1.0. Based on these assumed values, the error was calculated.

### 4.2. Proposed Model for Friction Correction–Compression Test

**Figure 12:**
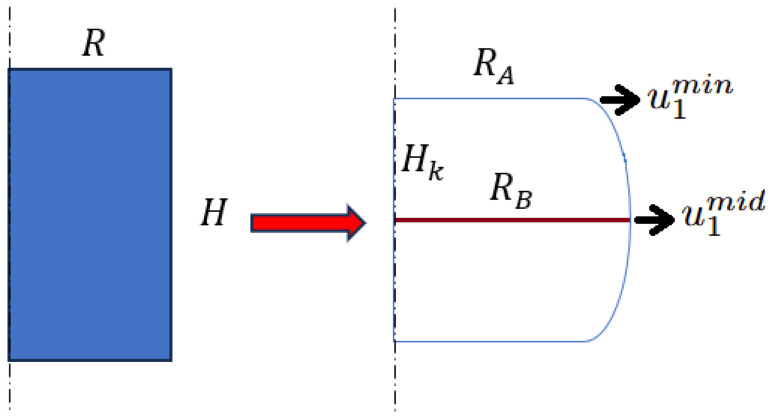
Shape of the specimen before and after the test; definition of barreling effect due to friction.

A new model is proposed for friction correction. This model allows estimating the friction coefficient with a small error and then using Equation (7) to calculate the correct stress level, $\sigma_{mat}$. Cylindrical specimens are used in the compression tests, both quasi-static and dynamic, as shown in Figure 12.

The initial radius of the specimen is *R* and its initial height is *H*. After compression, the final height of the specimen is $H_{k}$ and the plastic strain $\varepsilon_{\mathrm{pl}}$ can be calculated as follows:

(11) $$\varepsilon_{pl}=-\frac{H_{k}-H}{H}$$

For a positive friction coefficient during compression, the effect of barreling is visible in the specimen. The final radius of the specimen in the middle is $R_{B}$ and in the bottom and top is $R_{A}$. The relative barreling factor $B_{F}$ is calculated based on the following equation:

(12) $$B_{F}=\left(\frac{R_{B}-R_{A}}{R}\right)\cdot 100\%\;$$

Additionally, the specimen shape ratio $s_{0}$ is calculated based on the following equation, Equation (13):

(13) $$s_{0}=\frac{H}{2\cdot R}=\frac{H}{D}.$$

The relation between the relative barreling factor $B_{F}$ and the plastic strain $\varepsilon_{\mathrm{pl}}$ for two specimen shape ratios, $s_{0}=0.5$ and $s_{0}=1.0$, are analyzed.

Numerical simulations made it possible to calculate the curves for any friction coefficient between 0.05 and 0.15 in the $B_{F}$–$\varepsilon_{\mathrm{pl}}$ plane. Later, if these quantities are measured in actual experiments, the friction coefficient can be estimated. The following equation relates μ to $B_{F}$, $\varepsilon_{\mathrm{pl}}$, and $s_{0}$, as shown in Equation (14):

(14) $$\mu =\frac{B_{F}\left(\mu ,s_{0},\varepsilon_{pl}\right)}{s_{0}\;\left(p_{1}\varepsilon_{pl}+p_{2}\varepsilon_{pl}^{2}+p_{3}\varepsilon_{pl}^{3}+p_{4}\varepsilon_{pl}^{4}\right)}$$

**Figure 13:**
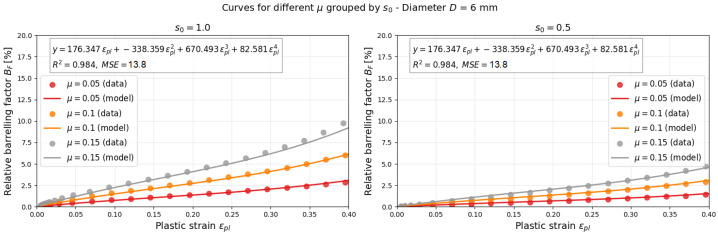
Global optimization results for *D* = 6 mm.

**Figure 14:**
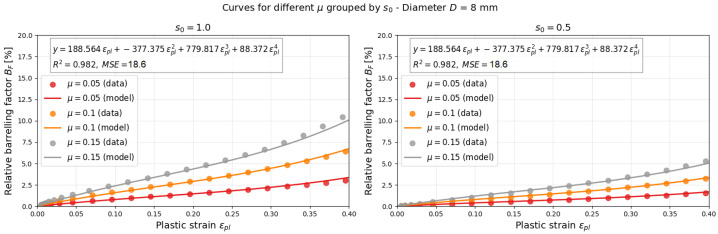
Global optimization results for *D* = 8 mm.

The parameters calculated using the least squares method are presented in Table 3 for two different diameters, *D* = 6 mm and 8 mm. The global optimization algorithm was used for both diameters. It includes results of the simulation for three friction coefficients, μ (0.05, 0.1 and 0.15), and two shape ratios, $s_{0}$ (1.0 and 0.5). Finally, the results of minimization of the error lead to the finding of the optimal parameters presented in Table 3. The model is optimized on the basis of simulation data of the dynamic compression tests. The visualization of the optimization process is finally presented in Figure 13 and Figure 14.

**Figure 15:**
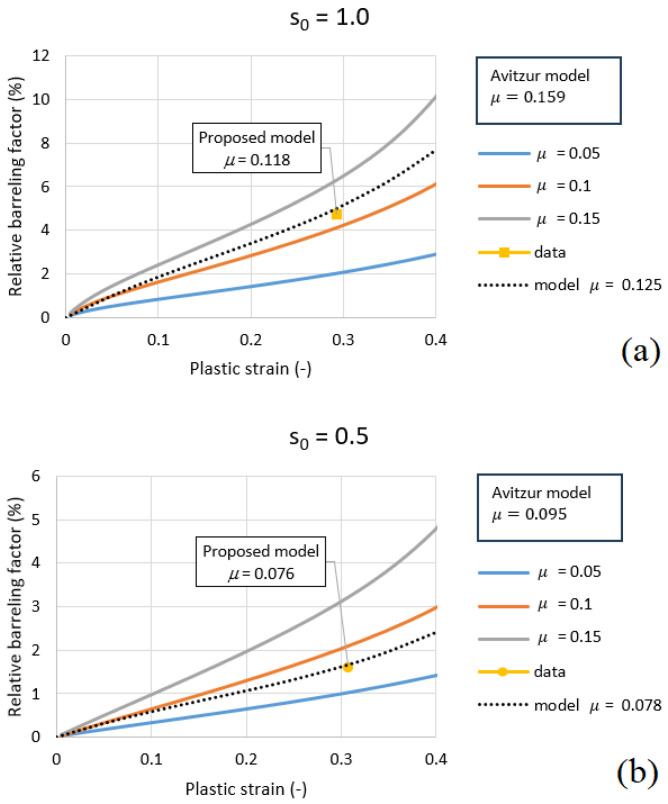
Relative barreling factor $B_{F}$ versus plastic strain $\varepsilon_{pl}$ for (**a**) $s_{0}$ = 1.0 and (**b**) $s_{0}$ = 0.5 (*D* = 6 mm).

**Figure 16:**
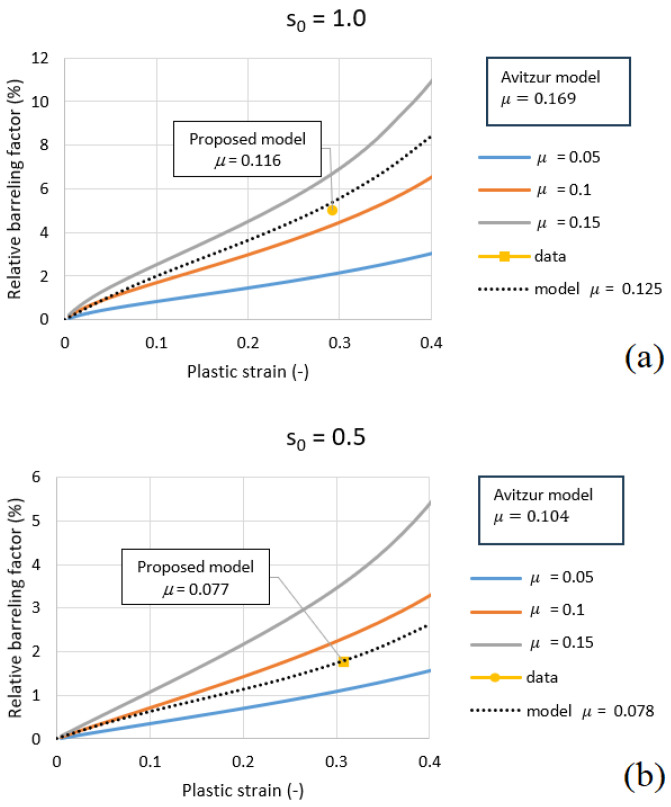
Relative barreling factor $B_{F}$ versus plastic strain $\varepsilon_{pl}$ for (**a**) $s_{0}$ = 1.0 and (**b**) $s_{0}$ = 0.5 (*D* = 8 mm).

The friction coefficient can be estimated based on the shape of the specimen after compression. According to the proposed model, the friction coefficients, μ, are 0.076 and 0.118 (D=6 mm) for $s_{0}=0.5$ and $s_{0}=1.0$, respectively. The values predicted by the model do not depend on *D* or *R*. For D=8 mm, the model predicts μ=0.077 and 0.116 for $s_{0}=0.5$ and $s_{0}=1.0$, respectively. The specimen diameter is only used to calculate $s_{0}$, as shown in Figure 15 and Figure 16. It is clear that the Avitzur model predicts higher friction coefficients, thus overestimating them: 0.095 and 0.104 for different *D* for $s_{0}=0.5$, and 0.159 and 0.169 for different *D* at $s_{0}=1.0$ (see Table 2).

Figure 15 and Figure 16 illustrates the variation in the relative barreling factor $B_{f}$ as a function of the plastic strain $\varepsilon_{pl}$ for two different geometries, (a) $s_{0}$ = 1.0 and (b) $s_{0}$ = 0.5, and two different diamterers, *D* = 6 mm and 8 mm. For both geometries, an increase in plastic strain leads to a progressive growth of the barreling factor, which becomes more pronounced at higher friction levels (μ = 0.15).

When comparing the proposed correction model with the Avitzur formulation, clear differences appear. For $s_{0}=1.0$, the Avitzur model predicts a friction coefficient of μ=0.104, while the proposed model gives a lower value, μ=0.077, closer to the assumed 0.078. Similarly, for $s_{0}=0.5$, the Avitzur model predicts μ=0.169, while the proposed model estimates μ=0.116, which is closer to the assumed 0.125. These results are valid for diameter D=8 mm, and the same trend is observed for D=6 mm (smaller sample).

These results confirm that the proposed correction model provides a more accurate estimate of the friction effect, particularly at higher plastic strains and higher friction levels. Consequently, this leads to more reliable predictions of the true stress–strain response in SHPB compression tests.

## 5. Conclusions

This study demonstrates that the precision and reliability of dynamic compression tests using the Split Hopkinson Pressure Bar (SHPB) are strongly dependent on both the geometry of the sample and the boundary conditions, particularly friction at the sample–bar interface. Friction was found to play a decisive role in shaping the stress distribution, influencing the onset of plastic deformation, and altering the measured strength of the materials. It promotes non-uniform stress states, causes barreling, and leads to overestimation of deformation forces, while also modifying failure mechanisms. Appropriate lubrication, careful surface preparation, and the application of finite element–based friction correction are essential to obtain the true properties of the material.

The geometry of the samples, expressed through the height-to-diameter ratio (*H*/*D*), was shown to govern the balance between the uniformity of stress and the magnitude of undesirable effects such as radial inertia, barreling, or buckling. Low *H*/*D* ratios improve stress homogeneity but increase radial inertia and barreling, while high *H*/*D* ratios reduce these effects but increase the risk of axial strain gradients and premature instability. The interaction between geometry and friction further amplifies measurement errors when the boundary conditions are not precisely controlled. Numerical simulations, particularly using Abaqus/Explicit, proved indispensable to identify these coupled effects, improve friction calibration curves, and validate new correction model.

The new friction correction method, based on the barreling factor and plastic strain, shows good agreement with measured friction coefficients (errors between 0.9 and 7.2%). It also indicates that the commonly used Avitzur model may overestimate friction by as much as 21 to 35%. The effects of friction are important. It changes stress distribution, causes barreling, increases apparent strength, and may influence failure modes. Geometry also plays a key role, since the height-to-diameter ratio (*H*/*D*) controls the balance between uniform stress and risks such as radial inertia or buckling. If not properly managed, the interaction between friction and geometry can increase measurement errors. Numerical modeling, especially finite element methods (FEMs), is essential for friction calibration, error evaluation, and building correction models. The new correction model based on the barreling factor gives more realistic friction estimates than the traditional Avitzur model.

This work investigated the influence of specimen geometry and friction on the accuracy of dynamic compression tests using the SHPB technique. The friction correction model is proposed and validated through numerical simulations data. The main findings are summarized as follows:

Scientific Contribution
  - A new friction correction model based on the *barreling factor* and plastic strain has been developed.
  - The model improves the accuracy of stress–strain measurements and enables more reliable identification of constitutive model parameters.
Improvements over Avitzur (Quantitative)
  - The proposed model reduces the friction coefficient error observed with the Avitzur approach from 21–35% to 0.9–7.2%.
  - It provides better agreement with experimental observations, particularly for short specimens and high-friction interfaces.
Practical Recommendations for SHPB Practitioners
  - For ductile materials, use low *H*/*D* ratios (0.5≤H/D≤1.0) but apply friction correction to account for barreling effects.
  - For brittle or high-strength materials, select higher *H*/*D* ratios (1.5≤H/D≤2.0) to minimize radial inertia and specimen instability.
  - The new correction model is recommended when accurate friction estimation is required, especially in cases involving high plastic strain or significant interface constraints.

## Figures and Tables

**Table 1:** Inconel 718 constants published in [24] used for the JC model, shown in Equation (6).

*A* (MPa)	*B* (MPa)	*n* (-)	*C* (-)	${\dot{\varepsilon }}_{0}$ (1/s)
400	1798	0.9143	0.0312	1

**Table 2:** Data used from simulation to calculate the friction coefficient based on the Avitzur and proposed models (dimensions and displacements are defined in mm).

$H_{0}$ or *H*	*R*	$s_{0}$	ΔH	$u_{1}^{\mathrm{mid}}$	$u_{1}^{\min}$	*H* or $H_{k}$	ΔR	*b* (Equation (9))	$\mu_{\mathrm{avit}}$ (Equation (10))	Error	$\varepsilon_{\mathrm{pl}}$ (Equation (11))	$B_{F}$ (Equation (12))	μ (Equation (14))	Error
3.00	3.00	0.5	0.9225	0.6203	0.5719	2.0775	0.0484	0.1453	0.095	**21.0 %**	0.3075	1.6133	0.076	**2.6%**
6.00	3.00	1.0	1.7750	0.6108	0.4688	4.2450	0.1421	0.4582	0.159	**27.2 %**	0.2925	4.7363	0.118	**5.3 %**
4.00	4.00	0.5	1.2300	0.8273	0.7566	2.7700	0.0707	0.1593	0.104	**33.5 %**	0.3075	1.7681	0.077	**0.9 %**
8.00	4.00	1.0	2.3400	0.8144	0.6147	5.6600	0.1997	0.4830	0.169	**35.0 %**	0.2925	4.9926	0.116	**7.2 %**

**Table 3:** Constants used for Equation (14) to define the friction coefficient value for two diameters *D*.

Parameters	*D* = 6 mm	*D* = 8 mm
$p_{1}$	176.347	188.564
$p_{2}$	−338.359	−377.375
$p_{3}$	670.492	779.817
$p_{4}$	82.581	88.372

**Figure A1:**
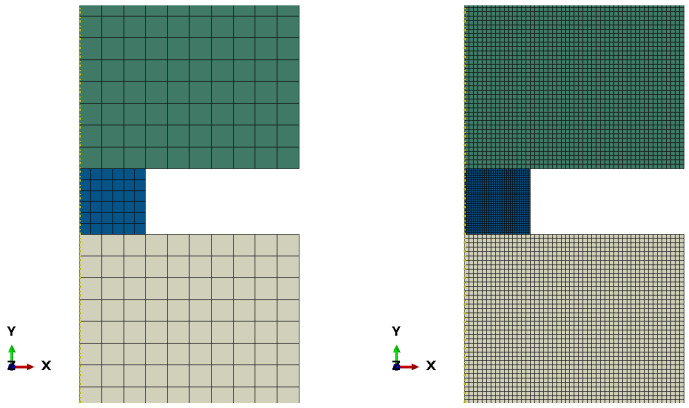
Zoomed-in image of the models with coarse (**left**) and fine (**right**) mesh.

**Figure A2:**
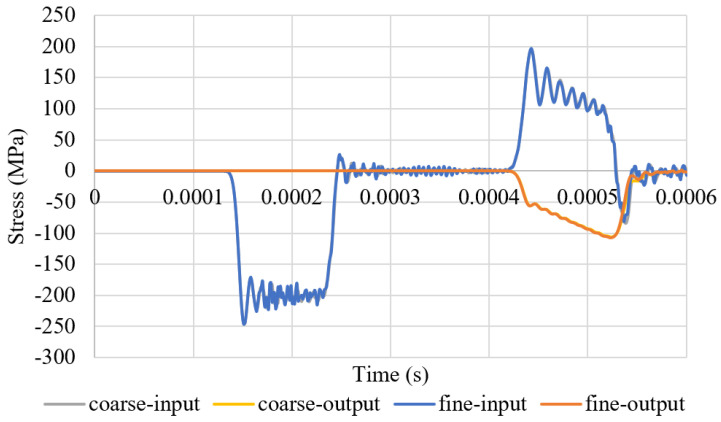
Mesh sensitivity on both signals (input and output).

**Figure A3:**
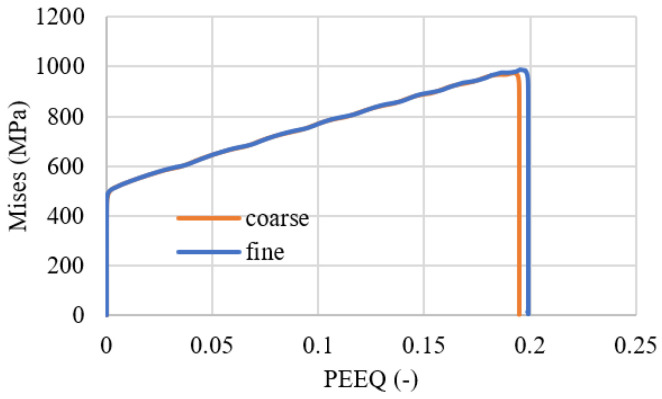
Stress–strain curves for both meshes.

**Figure A4:**
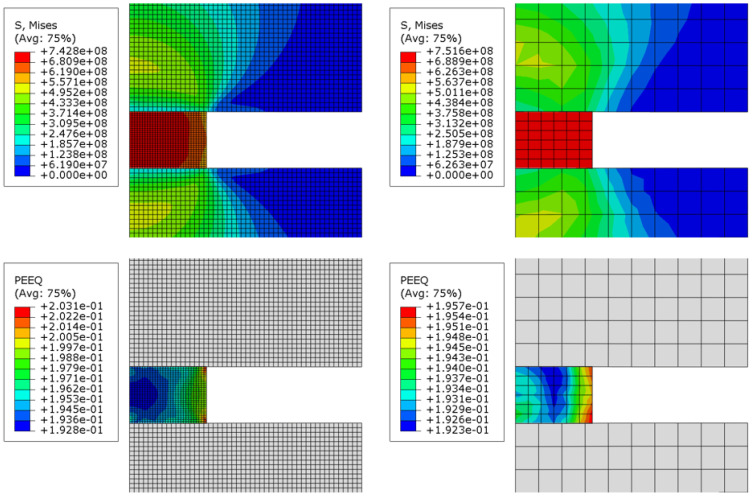
Distribution of Mises stress in MPa and equivalent plastic strains (PEEQs).

## Data Availability

The original contributions presented in this study are included in the article. Further inquiries can be directed to the corresponding author.

## Appendix A. Mesh Size Sensitivity

In finite element analysis, the size of the mesh is critical, as it affects accuracy and the number of elements required [23,27]. The optimal mesh size should be analyzed every time before presentation of the results. In this paper, it was also considered (Figure A1). There are two meshes: (1) coarse (on the left) with 0.0005 m mesh size in the specimen and 0.001 m in the bars and projectile and (2) fine (on the right) with 0.0001 m in the specimen and 0.0002 m in the bars and projectile. We consider a five-time denser mesh in the case of a fine mesh.

The longitudinal stress waves are calculated and recorded in the middle of the bars and are presented in Figure A2 for two considered meshes. The waves are very similar, and finally the stress–strain curves calculated for both signals are also in agreement, as presented in Figure A3.

Additionally, the distribution of Mises stress and equivalent plastic strain is presented at the end of the loading before the specimen is unloaded. At this point, the stress and strain have maximal values.

In conclusion, the mesh size sensitivity is a fundamental consideration in finite element modeling that affects both the accuracy and computational efficiency of simulations. The literature consistently highlights the need for careful mesh design to avoid inaccuracies and ensure reliable results.
